# Characterization of *Klebsiella pneumoniae* isolated from patients suspected of pulmonary or bubonic plague during the Madagascar epidemic in 2017

**DOI:** 10.1038/s41598-022-10799-4

**Published:** 2022-04-27

**Authors:** Andriniaina Rakotondrasoa, Lova Maminirina Andrianonimiadana, Soloandry Rahajandraibe, Solohery Razafimahatratra, Voahangy Andrianaivoarimanana, Soanandrasana Rahelinirina, Tania Crucitti, Sylvain Brisse, Victor Jeannoda, Minoarisoa Rajerison, Jean-Marc Collard

**Affiliations:** 1 Experimental Bacteriology Unit, Institut Pasteur Madagascar, Antananarivo, Madagascar; 2 Plague Unit, Institut Pasteur Madagascar, Antananarivo, Madagascar; 3 Biodiversity and Epidemiology of Bacterial Pathogens, Institut Pasteur, Paris, France; 4 Mention Biodiversité Et Santé, Sciences Faculty, University of Antananarivo, Antananarivo, Madagascar; 5 Present Address: Experimental Bacteriology Laboratory, Center for Microbes, Development and Health (CMDH), Institut Pasteur of Shanghai/Chinese Academy of Sciences, Shanghai, People’s Republic of China

**Keywords:** Microbiology, Molecular biology, Diseases, Medical research

## Abstract

*Klebsiella pneumoniae* can lead to a wide range of diseases including pneumonia, bloodstream and urinary tract infections. During a short period of a pulmonary plague epidemic in October 2017 in Madagascar, 12 K*. pneumoniae* isolates were identified in ten sputum and two buboes aspirate samples. These isolates were from 12 patients suspected of plague, without epidemiological relationships, but were negative for *Yersinia pestis* in culture. Data were collected from the plague national surveillance system. The isolates were characterized by antimicrobial susceptibility testing and whole-genome sequencing. Real-time PCR was performed to confirm the presence of *K. pneumoniae* DNA in buboes. All isolates were identified as *K. pneumoniae* sensu stricto. Five isolates were extended-spectrum β-lactamases producers; eleven different sequence types were identified. Five isolates belonged to known hypervirulent sequence types. Our results demonstrate community-acquired pneumonia caused by *K. pneumoniae* isolates in patients suspected of plague stressing the importance of bed-side differential diagnosis.

## Introduction

Between August 1st and November 26th, 2017, a total of 2414 clinically suspected plague cases were reported to the Central Laboratory for Plague (CLP) at the Institut Pasteur de Madagascar, including 1878 (78%) pulmonary plague (PP), 395 (16%) bubonic plague (BP), one (< 1%) septicaemia and 140 (6%) cases with unspecified clinical form^1^. This predominantly urban plague epidemic was characterised by a large volume of notifications in two major urban areas (Antananarivo and Toamasina) and by an unusually high proportion of pneumonic forms. According to the 2006 WHO standard plague case definitions and using the results of three types of diagnostic tests assessed (rapid F1-antigen diagnostic test, (RDT), molecular amplification method, and culture)^2^, 386/1,878 (21%) were probable and 32/1,878 (2%) were confirmed cases among the notified PP cases. The magnitude of this PP outbreak is likely to have been smaller than suggested by notified suspected cases^1^; and its severity indicated by the case fatality rate among confirmed plus probable cases (about 9%) was substantially lower than observed in the last previous 18 years (25%)^3^. Over-reporting of PP cases due to limited clinical experience in the two most affected areas, and the difficulty to clinically diagnose PP through respiratory signs was speculated. The clinical diagnosis of PP from polymicrobial sputum associated with other potential causes of pneumonia remains a challenge because the isolation of *Yersinia pestis* (*Y. pestis*) is more complicated compared to other bacteria.

*Klebsiella pneumoniae (K. pneumoniae)* is a Gram-negative bacterium naturally resistant to amoxicillin and carbenicillin. *K. pneumoniae* complex members comprise 7 phylogroups (Kp1 to Kp7) that have been given taxonomic status as *K. pneumoniae* sensu stricto, *K. quasipneumoniae* subsp. *quasipneumoniae*, *K. quasipneumoniae* subsp. *similipneumoniae*, *K. variicola* subsp. *variicola*, *K. variicola* subsp. *tropica*, ‘*K. quasivariicola’*, and *K. africana*, respectively^4^. *K. pneumoniae* sensu stricto has become an important multidrug resistant pathogen of the last decade with multiple resistance determinants, mostly for aminoglycosides, cephalosporins and carbapenems^5^. It is commonly isolated from hospital-acquired infections including pneumonia, bloodstream infection, urinary tract infection, and community acquired infections such as pyogenic liver abscess, meningitis and pneumonia. The capsule is an important virulence factor that protects *K. pneumoniae* from phagocytosis, with over 79 defined capsular serotypes. Isolates with K1 and K2 capsular serotypes are associated with virulent infections. However, not all K2 capsular isolates are virulent^6^. Virulence factors associated with hypervirulent *Klebsiella* infections also include siderophores, including aerobactin and salmochelin, which are typically encoded on virulence plasmids; yersiniabactin (typically chromosomally encoded in an integrative and conjugative element), and the hypermucoviscosity factor *rmpA* gene (also typically on the virulence plasmid)^7–12^.

Differential diagnosis is important and should be included in the diagnostic procedures in order to detect and identify other pathogens among the suspected but not confirmed cases of plague. *K. pneumoniae* is one of the pathogens that cause severe bacterial lung infections and which should be considered in non-confirmed suspected pulmonary plague cases. However, *K. pneumoniae* was not considered among suspected bubonic plague cases^13^.

The purpose of this study was to characterize *K. pneumoniae* isolates from some of clinically suspected plague patients during the plague outbreak in Madagascar in 2017. We aimed to analyze their population structure and clonal diversity using core genome Multilocus Sequence Typing (cgMLST). Further, our aims were to analyze their resistance and virulence genes, and the association of virulence factors and phenotype with clonal background.

## Material and methods

### Patients and bacterial isolates

Patients with confirmed *K. pneumoniae*, isolated during nine days of the plague epidemic (from the 6th of October 2017 till the 14th of October 2017), were included in this sub-study. Epidemiological, clinical and lab data of patients were extracted from the plague national surveillance system database of Institut Pasteur de Madagascar between August 1st and November 26th in 2017^1^. *Y. pestis* was isolated from biological samples (bubo aspirates for BP, sputum for PP) by direct culture on *Yersinia* selective Cefsulodin-Irgasan-Novobiocin (CIN) agar medium (Oxoid Ltd., United Kingdom) and *Y. pestis* detection by PCR was performed on all samples. All methods were carried out according to the 2006 WHO recommendations^14^. Culture incubation was done at 26–28 °C for 48 h or longer as *Y. pestis* grows slower than other bacteria. Colonies obtained within 24 h on CIN medium and which did not have *Y. pestis* morphology were identified on MALDI-TOF MS (Biotyper version 3.3, Bruker Daltonics, Champs-sur-Marne, France). Colonies identified as *K. pneumoniae* were further purified on Simmons Citrate Agar Inositol (SCAI) medium^15^. The Central Laboratory for Plague of the Malagasy Ministry of Health is hosted at the Plague Unit-WHO Collaborating Centre of the Institut Pasteur and all methods used in this study were performed in accordance with the relevant guidelines and regulations. The data reported here are parts of the plague national surveillance system and no specific additional ethics approval was necessary. All information on individual patients has been anonymized for presentation.

### Phenotype detection

The hypermucoviscosity phenotype of the *K. pneumoniae* isolates was determined using the string test, in which a standard bacteriological loop is used to stretch a mucoviscous string from each colony cultured on SCAI. The formation of a viscous string > 5 mm in length was regarded as a positive test result^12^. The string test results were confirmed using colonies grown on blood agar.

### Bacterial susceptibility testing

Antibiotic resistance profiles were determined by the standard disc diffusion method according to CASFM-EUCAST V2-0-May2017 guidelines and using breakpoints for *Enterobacteriaceae*^16^ (http://www.sfm-microbiologie.org). Isolates were tested against 17 commonly used antimicrobial agents, namely amoxicillin, amoxicillin-clavulanate, piperacillin/tazobactam, cefalotin, cefoxitin, cefotaxime, ceftazidime, cefepime, aztreonam, imipenem, ertapenem, tobramycin, gentamicin, nalidixic acid, ciprofloxacin, trimethoprim-sulfamethoxazole and tetracycline. In addition, extended spectrum β-lactamase (ESBL) production was tested using the standard double disc synergy test.

### Genome sequencing and analysis

Genomic DNA of the *K. pneumoniae* isolates was extracted using DNeasy Blood & Tissue kit (Qiagen, Germany) and was subjected to whole genome sequencing. Genomic libraries were constructed using the Nextera XT DNA library preparation kit with dual indexing (Illumina, San Diego, USA). The libraries were sequenced on an Illumina NextSeq-500. Genome assembly was performed de novo using Spades Genome Assembler (Version 3.10.0). Genome analyses for core genome MLST (cgMLST) on 632 core genes were performed^17^. Sequence types (ST) were determined with an in silico MLST pipeline that assembles and compares sequences against allele data derived from the public MLST database at https://bigsdb.pasteur.fr/. Virulence genes and capsular serotypes (K-types) were assigned using the *K. pneumoniae* database hosted through the BIGSdb web application of the Institut Pasteur in Paris (https://bigsdb.pasteur.fr/klebsiella). Antimicrobial resistance genes were identified from genome sequences using the Resfinder (version 3.2)^18^. Plasmid replicon types were determined by using the Plasmidfinder (version 2.0)^18^ tool at https://genomicepidemiology.org. Parsnp was used for the core genome alignment^19^, while the Gubbins^20^ software tool was used to remove the single nucleotide polymorphisms (SNPs) from recombined regions and to create a refined phylogenetic tree; this tool uses RaxML^21^ to build the maximum likelihood phylogenetic tree on the recombination-free regions. The tree was subsequently annotated with iTOL (http://itol.embl.de/itol.cgi)^22^.

### Buboes K. pneumoniae screening by real-time PCR

We performed a real-time PCR targeting the *zur-khe* intergenic region (called the ZKIR qPCR assay)^23^ on the bubo samples in order to confirm the presence of *K. pneumoniae* DNA in the bubo and to exclude any technical contamination during culture. Bacterial DNA was extracted from the bubo samples using DNeasy Blood & Tissue kit (Qiagen, Germany). The real-time PCR assay was performed as previously described with the difference that we used 10 µl of SsoAdvanced universal SYBR Green Supermix (Bio-Rad, USA)^23^. Amplifications were performed using the CFX-96 (Bio-Rad, USA) platform. The positive controls consisted of DNA from *K. pneumoniae* UAA2239 and UAA2016 which are reference strains from the National Reference Center for Antibiotics from the Institut Pasteur in Paris, the negative control was plain molecular grade water.

### Ethics statement

The Ethics Committee/IRB authorized the use of the patient samples in this study, as long as they are anonymised/de-identified (reference number 261 MSANP/SG/AMM/CERBM).

No additional data was collected.

All patients provided oral consent and voluntarily agreed for sampling for diagnostic purposes.

### Nucleotide sequence accession numbers

WGS data have been deposited at the National Center for Biotechnology Information (NCBI) under BioProject PRJNA565154.

## Results

### Case presentation and K. pneumoniae antimicrobial susceptibility

Twelve clinical samples (2 bubo aspirates for BP, 10 sputum for PP) out of 496 collected between 06 and 14 October 2017 in Antananarivo (N = 362) and Toamasina (N = 134) screened for *Y. pestis* presence by culture on CIN medium gave rise to abundant colonies (> 10^3^ CFU/ml) with a typical *K. pneumoniae* morphology (moist, dome-shaped) after 24 h incubation. One representative isolate per plate was selected and the twelve isolates were identified as *K. pneumoniae* by MALDI-TOF MS. Using PCR, *Y. pestis* DNA was not detected in samples. We did not perform serology for antibody detection in the confirmed and suspected cases. This period was reported as the peak of the plague epidemic curve, with essentially PP cases. Patients had early clinical signs suggesting pulmonary, secondary pulmonary or bubonic plague in an epidemic setting.

The samples containing *K. pneumoniae* were 10 sputum samples and two bubo aspirates and were negative for *Y. pestis* colonies. The description of the 12 patients is shown in Table 1. There was no mortality among these patients (Table 1). Two-thirds of the patients (N = 8) were from Antananarivo and one-third (N = 4) was from Toamasina. Seven patients were men. Five patients were younger than 18 years, and six were 19 to 27 years old and one was 46 years old. Fever status was reported for 10 patients, eight of them had body temperatures > 37.5 °C. According to the clinical forms, nine patients were suspected of having PP, one was defined to suffer from secondary PP, one patient was suspected of BP and for another patient, data about the clinical form was lacking.

**Table 1:** Study population.

Isolates	Sites	Samples	Age	Sex	Profession	T°	Clinical form	*Bubo size	State of health	C^a^	BS^b^	CP^c^
PP1	Antananarivo	Sputum	15	Female	Pupil	38	Pulmonary plague	–	G^d^	1	0	1
PP2	Antananarivo	Cervical bubo	3	Female	NA	37.8	Bubonic plague	3	G	0	0	0
PP3	Antananarivo	Sputum	19	Male	Unknown	38	Pulmonary plague	–	G	1	0	1
PP4	Antananarivo	Sputum	20	Male	Unknown	38.1	Pulmonary plague	–	G	1	0	1
PP5	Antananarivo	Sputum	1	Female	NA	38.7	NA	–	W	0	0	0
PP6	Antananarivo	Sputum	1	Male	NA	39	Pulmonary plague	–	W	1	0	0
PP7	Antananarivo	Sputum	19	Male	Military	37	Pulmonary plague	–	W^e^	1	1	0
PP8	Toamasina I	Bubo	17	Female	NA	–	Pulmonary plague II	2	G	1	0	0
PP9	Toamasina I	Sputum	21	Female	Student	38	Pulmonary plague	–	G	1	1	0
PP10	Toamasina I	Sputum	27	Male	Physician student	37.1	Pulmonary plague	–	G	1	0	1
PP11	Toamasina I	Sputum	20	Male	Electronics	37.5	Pulmonary plague	–	W	1	0	0
PP12	Antananarivo	Sputum	46	Male	Journalist	–	Pulmonary plague	–	G	0	0	1

*C*^*a*^ cough, *BS*^*b*^ bloody sputum, *CP*^*c*^ chest pain, *G*^*d*^ Good, *W*^*e*^ Weak, *NA* not available, *3 size of a lychee core, *2* size of a bean.

Four of the patients coughed for at least 5 days and complained of chest pain, although they were in an overall good state of health. Two patients had signs of hemoptysis, and one of them was in weak health. Two patients coughed without further complaints but one was in weak health.

Four patients were under antibiotic treatment at the time of sample collection: two with trimethoprim-sulfamethoxazole; one with doxycycline and one with gentamycin. One patient who received trimethoprim-sulfamethoxazole was treated in addition with amoxicillin.

**Figure 1:**
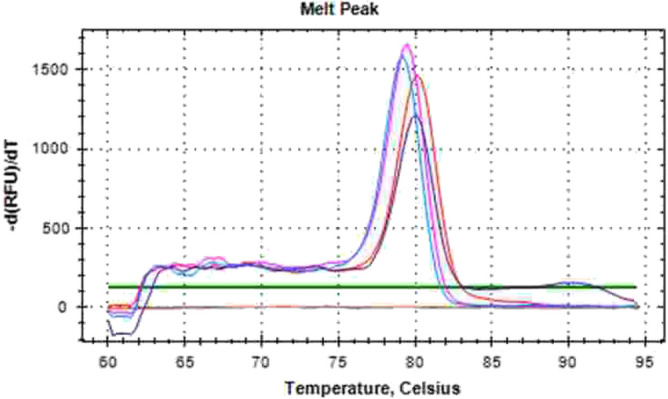
Melting curve results from ZKIR region detection obtained after 40 cycles using the ZKIR quantitative PCR system. DNA from two positive controls from *K. pneumoniae* strains (blue: UUA2239, violet: UUA2016). DNA extracted from patients’ buboes is in pink for PP2 and in red for PP8. Orange color corresponds to negative control.

The presence of *K. pneumoniae* DNA in the two bubo samples was also detected by real time-PCR. Melting curve values for the detection of *K. pneumoniae* were 79 °C and 80 °C for the positive controls and the DNAs extracted from buboes, respectively (Fig. 1). Of the 12 isolates, four had a positive string test. Five isolates were ESBL producers. Six isolates were resistant to sulfonamides and trimethoprim. Three and two isolates were resistant to gentamycin and tobramycin, respectively. One isolate was resistant to ciprofloxacin (Table 2).

**Table 2:** MLST profiles, cps K-types, resistance profile, resistance genes and virulence genes.

Isolates	Phylogroups	String test	ST	K_type	Resistance profile	Resistance genes/	Virulence genes
						Incompatibility group	
PP1	Kp1		280	KL23	AMX, AMC, TZP, CEF, CTX, CAZ, FEP, ATM, TOB, GEN, CIP, SXT, TET	*qnrB66, aac(3)-IIa, bla*_CTX-M-15_*, bla*_SHV-27_*, bla*_TEM-1B_*, dfrA14, strA, strB, sul2, tet(A) /* IncFIB(K), IncFII(K)	*mrk*
PP2	Kp1		3441	ND	AMX, AMC, CEF, CTX, CAZ, FEP, ATM, ETP, SXT, TET	*bla*_CTX-M-15*,*_* bla*_SHV-1_	*kfuA, mrk*
PP3	Kp1	+	380	KL2	AMX, AMC, TET	*bla*_SHV-1_	*clb, fyuA, iroBCDN, irp1/2, kfuC, kvgA, mrk, ybt*
PP4	Kp1		3442	KL1	AMX, AMC, CEF, CTX, CAZ, FEP, ATM, TOB, GEN, SXT, TET	*qnrB66, aac(3)-IIa, bla*_SHV-27_*, tet(A) /* IncFIB(K)	*fyuA, irp1/2, mrk, ybt*
PP5	Kp1		23	KL1	AMX, AMC, TET	*bla*_SHV-108_	*clb, fyuA, kfuA, mrk, allS, fdrA, gcl, glx, hyi, irp1/2, ybt*
PP6	Kp1		327	ND	AMX, AMC, CEF, CTX, CAZ, FEP, ATM, ETP, TOB, GEN, NAL, SXT	*aac(3)-IIa, bla*_SHV-101_	*kfu, mrk*
PP7	Kp1		3012	ND	AMX, AMC, SXT,TET	*bla*_SHV-11_	*mrk*
PP8	Kp1		716	ND	AMX, AMC, CEF, CTX, CAZ, FEP, ATM, ETP, CIP, SXT, TET	*bla*_SHV-27_	*mrk*
PP9	Kp1	+	86	KL2	AMX, AMC	*bla*_SHV-1_ */* IncHI1B	*iroBCDN, iucABCD, kvgA, mrk*
PP10	Kp1		65	KL2	AMX, AMC, TET	*bla*_SHV-11_ */* IncHI1B	*clb, mrk, iucABCD*
PP11	Kp1	+	3443	KL2	AMX, AMC	*bla*_SHV-1_ */* IncHI1B	*iucABCD, kvgS, mrk*
PP12	KP1	+	86	KL2	AMX, AMC	*bla*_SHV-1_	*mrk*

*ND* not determined.

### Genome analysis

**Figure 2:**
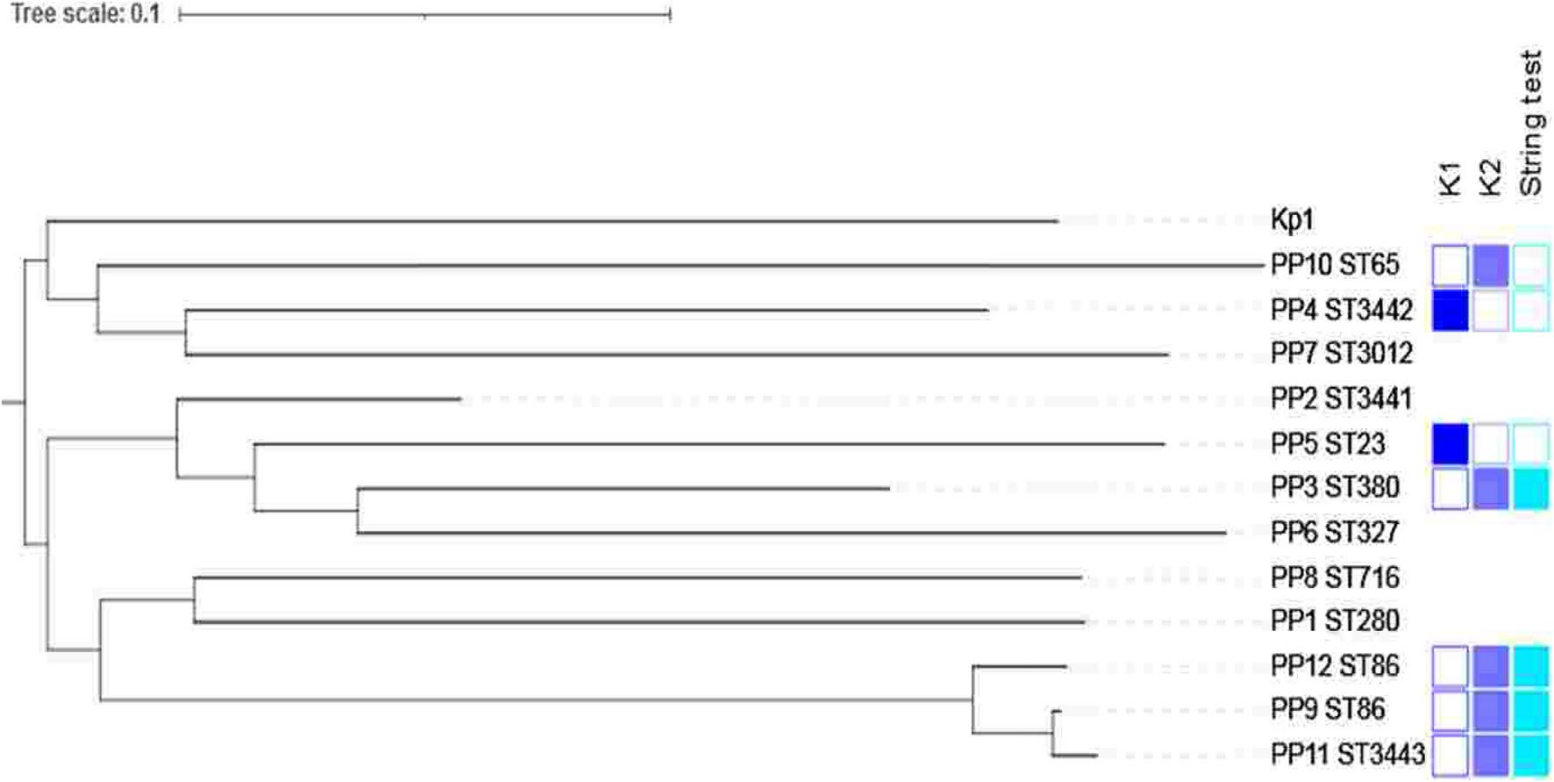
Phylogenetic tree of *K. pneumoniae* isolates obtained from 12 patients. The tree includes the sequence types (ST) of each isolates, the frequency of K-types in indigo and the string test positive in blue sky.

Whole genome sequencing of the *K. pneumoniae* isolates allowed us to characterize cgMLST alleles, virulence genes, capsular loci and resistance genes. All *K. pneumoniae* isolates were *K. pneumoniae* sensu stricto *(*Kp1) (Fig. 2). The 12 isolates had 11 different sequence types (STs): ST23 (N = 1); ST86 (N = 2); ST65 (N = 1); ST280 (N = 1); ST327 (N = 1); ST380 (N = 1); ST716 (N = 1); and ST3012 (N = 1), and three new STs: ST3441 (N = 1), ST3442 (N = 1), ST3443 (N = 1) (Table 2). Comparative genomic analysis of the two Kp ST86 isolates showed that they differed from each other by 123 alleles out of 632 scgMLST gene loci and are therefore unrelated epidemiologically.

The virulence genes identified in most isolates corresponded to the colibactin locus (*clb*), siderophores (*iroBCDN*), iron uptake systems and regulators (*kfu*, *kvgA*, respectively*)*, and yersiniabactin (*fyuA*, *irp1/2* and *ybt*) (Supplementary data). The PP3, PP9, PP11 and PP12 isolates were positive for the string test. These isolates belonged to ST320 (PP3), ST86 (PP9 and PP12), and ST3443 (PP11). All the ST23, ST65, ST86 and ST380 isolates had the gene *rmpA* associated with the hyperproduction of the capsule and also carried the *iucABCD* genes coding for the synthesis of aerobactin (Supplementary Data).

Isolates belonging to ST23, ST65, ST86 and ST380 were susceptible to all antibiotics tested, with the exception of amoxicillin to which *K. pneumoniae* is intrinsically resistant.

A total of five isolates were ESBL producers (Table 2). The ST280 and ST3441 isolates carried the *bla*_CTX-M-15_ gene. The ST3442-KL1 isolate harboring virulence genes (*fyu*, *irp1/2*, *ybt*) was ESBL producer and carried the cassette comprising *qnrB66*, *aac (3) -IIa*, *bla*_SHV-27_ and *tet* (A), as well as the IncFIB_K_ replicon marker (Table 2).

## Discussion

Unexpectedly, 12 cases of *K. pneumoniae* infection were detected and identified among individuals clinically suspected to have plague. No *Y. pestis* was identified from their clinical samples, but three patients (PP8, PP10 and PP11) yielded a positive result on RDT. However, culture is the gold standard for the identification of *Y. pestis* and RDT could provide false results^24^. All patients had no epidemiological relationships and no family member or contact had been recorded with plague.

Although the selective medium for *Yersinia* was not intended for *K. pneumoniae* isolation, *K. pneumoniae* does grow on the CIN medium in 24 h. The selectivity of this medium is reported as being partial, as other Gram-negative bacilli can grow on CIN medium, including other species of Enterobacterales able to ferment mannitol^25^. Therefore, full species identification is recommended.

It is not surprising to isolate *K. pneumoniae* from pneumonia cases. Community-acquired *K. pneumoniae* infections are common, including in Africa^26,27^. In contrast, to our best knowledge, *K. pneumoniae* isolated from buboes aspirates were never reported previously. Additionally to culture, the presence of *K. pneumoniae* in buboes aspirates was confirmed by PCR. The advantage of melting curve analysis over Taqman based real time-PCR is its lower costs without losing specificity^28^. Further studies are needed to evaluate this method, which could be used in screening for *K. pneumoniae* in buboes or other suspected biological samples.

*K. pneumoniae* has the capacity to acquire resistance genes and to become increasingly more difficult to treat. One of the *K. pneumoniae* isolates detected in one of two patients who were treated by combination of amoxicillin and trimethoprim-sulfamethoxazole developed resistance. However, *K. pneumoniae* is known to be intrinsically resistant to ampicillin due to the presence of the chromosomal β-lactamase genes *bla*_SHV-1_, *bla*_SHV-11_ or similar^29^. At the same time, we identified in this strain (ST280), the two genes *dfrA14* and *sul2*, associated to resistance to trimethoprim and sulfamethoxazole, respectively. In addition, among all isolates, it was the only *K. pneumoniae* isolates to be resistant to piperacillin/tazobactam (TZP), which is concordant with the presence of the *bla*_TEM-1B_ gene^30^.

Two of the five ESBL isolates harbored *bla*_CTX-M15_. This gene was commonly found in ESBL-producing *K. pneumoniae* isolated in Madagascar^26,27^. *bla*_SHV27_ and *bla*_SHV101_ were found in the other ESBL-producer.

According to MLST analysis, we observed the presence of five STs known to be associated with hypervirulence, including STs ST23 (N = 1), ST65 (N = 1), ST86 (N = 2) and ST380 (N = 1). In addition, a *K. pneumoniae* isolate having the new ST3443 differs by a single locus from ST86 on the *tonB* locus (allele 18 instead of 27). Whole genome analysis of *K. pneumoniae* showed that the isolates with common ST differed from each other by alleles occurring outside the 7 household genes. The strain ST3443 was tested string positive, as was also the case for the ST380 strain and the two ST86 strains. The presence of common virulence genes in the ST23 isolate which were ICEKp10 encoding *clb 2* sequence variants, *ybt 1* and *rmpA/rmpA2* suggests its belonging to CG23 sublineage I (CG23-I)^31^.

MLST typing of *K. pneumoniae* isolated in different countries revealed that ST23, ST65, ST86 and ST380 were responsible for pyogenic liver abscess cases and other invasive community-acquired infections^32^. These isolates have been reported particularly in Asia, but their diffusion outside Asia has been described^31^. Among virulence factors, *rmpA* and aerobactin are the most important ones^33^. The presence of genes responsible for the hypermucoviscosity phenotype, notably *rmpA*, plays an important role in the virulence of *K. pneumoniae* isolates. This gene is often associated with serotype K1 and K2. Expression of *rmpA* allows the bacteria to escape the host's defense system and colonize the mucous membranes. Epidemiological studies have shown that the majority of ST23 are related to K1 capsular serotypes and liver abscesses^31,34^, while K2 is the second capsular serotype resulting in community-acquired pneumonia^33^. Yersiniabactin, a virulence gene (*Ybt*), detected in the three *K. pneumoniae* isolates serotyped KL1 has been reported as the iron absorption system in highly virulent *Y. pestis*^35^, and was later shown to have evolved ancestrally within the *Klebsiella* genus^25^. Several studies have shown isolates belonging to these STs (23, 65, 86 and 380), with the same combination of virulence factors, to be virulent in mouse models^12,31^.

During an epidemic, knowledge of the etiology is essential in order to provide the most adapted treatment to patients. Microbiological diagnosis can improve the effectiveness of treatments, avoid long-term complications for the infected patient, and in addition avoid widespread overuse and misuse of antibiotics. Early diagnosis can help to prevent or stop an outbreak too. One of the reasons for a possible treatment failure could arise during inaccurate diagnoses and inappropriate treatments. Similar symptoms can lead to routine treatments based on syndromic approaches which are often applied in developing countries, hence the importance of including differential diagnosis in laboratory diagnostic procedures in order to identify the etiology. As the physician is rarely able to make an etiological diagnosis on clinical grounds alone, treatment should ideally be based on the result of bacteriological examination. In this case, bacteriological diagnosis could be complicated by the fact that the respiratory tract could be infected by *K. pneumoniae*^36^. Although the population we included in our study is young, the clinical signs of a few patients warned us of possible serious infections due to *K. pneumoniae* such as bloody sputum and a chest pain which were among typical signs of pestis pneumonia. However, *Y. pestis* was not found in culture*.* Typically, the plague is better known by its three clinical forms: bubonic, septicemic and pulmonary plague while hypervirulent *K. pneumoniae* strains are known to cause pneumonia, sepsis, liver abscesses and meningitis^37–39^.

We acknowledge the following limitations of our study. First, we studied a limited number of samples in a short duration of the epidemic, which is far from being representative of all the negative samples for *Y. pestis*. Second, detailed data about clinical characteristics and outcomes were lacking due to the outbreak emergency context. Finally, we did not confirm the virulence of the *K. pneumoniae* strains using mouse models.

## Conclusions

Although few samples were studied, within a short duration of inclusion (9 days from 06/10/17 to 14/10/17), our results show that some plague-suspected patients in fact acquired a pneumonia caused by *K. pneumoniae*. Bacterial identification proved useful for determining the etiology. WGS and AST results showed that among the 12 K*. pneumoniae* isolates, there were ESBL producers and virulent strains. This study reports on the genomic characterization of *K. pneumoniae* isolates isolated from patients during an epidemic of plague. These results could serve to warn clinicians regarding the most adequate treatment. Likewise, we reported the importance of bacteriological diagnosis for improving patient management.

## Supplementary Information

Supplementary Information.
